# Development of a gene-activated matrix for enhanced AAV gene delivery *in vitro*

**DOI:** 10.3389/fbioe.2026.1832901

**Published:** 2026-06-10

**Authors:** Ahmed Musoski, Florent Poulhès, Cedric Sapet, Neelam Iqbal, Payal Ganguly, Maximilian Kampick, Bastian Hoechst, Mario Marotta, Katja Dumler, Animesh Jha, Elena A. Jones, Peter V. Giannoudis, Marilys Blanchy, Percy Knolle, Olivier Zelphati, Martina Anton

**Affiliations:** 1 Institute of Molecular Immunology, TUM University Hospital Rechts der Isar, School of Medicine and Health, Technical University of Munich (TUM), Munich, Germany; 2 OZ Biosciences, Marseille, France; 3 School of Chemical and Process Engineering, University of Leeds, Leeds, United Kingdom; 4 Leeds Institute of Rheumatic and Musculoskeletal Medicine, University of Leeds, Leeds, United Kingdom; 5 NIHR – Leeds Biomedical Research Centre, Leeds Teaching Hospital Trust, Leeds, United Kingdom; 6 Leitat Technological Center, Terrassa, Spain; 7 Academic Department of Trauma and Orthopaedic Surgery, School of Medicine, University of Leeds, Leeds, United Kingdom; 8 APPLUS Rescoll, Pessac, France

**Keywords:** adeno-associated viral vectors, bone regeneration, chitosan tricalcium phosphate, gene-activated matrix, poloxamer

## Abstract

Recombinant AAV vectors are among the most extensively studied vectors for viral gene delivery due to their unique safety profile and their ability to mediate efficient, long-term transgene expression by persisting episomally in the nucleus. These properties make AAV vectors promising not only for the treatment of monogenic diseases but also for tissue regenerative applications. In the context of critical-sized bone defects, current gold-standard treatments are often associated with severe side effects, highlighting the need for alternative therapy strategies. In this study, we therefore developed a gene-activated matrix (GAM) for localized AAV-mediated gene delivery for potential applications in bone regeneration, establishing a workflow that is straightforward and transferable to other therapeutic settings. Following an initial screening of AAV serotypes and transgene DNA formats, reporter gene–expressing AAV2 vectors were associated with chitosan-based scaffolds containing varying amounts of β-tricalcium phosphate (β-TCP). Analysis of AAV release revealed that incorporation of β-TCP significantly reduced AAV release from 15.7% to approximately 6.6%. Furthermore, seeding of primary ovine mesenchymal stromal cells (oMSC) onto AAV-loaded scaffolds demonstrated efficient *in situ* delivery and expression of the osteogenic and angiogenic growth factors BMP-2 and VEGF *in vitro*. To further enhance AAV-mediated gene delivery, a panel of poloxamers was screened, leading to the identification of novel transduction enhancer AAVBlast. AAVBlast stabilized AAV particles and increased their bioavailability, resulting in significantly elevated intracellular AAV DNA levels, enhanced transgene mRNA expression, and increased protein production across multiple cell types. Modular application of AAVBlast onto the GAM significantly enhanced transduction of scaffold-released AAV particles but did not significantly affect transduction of oMSC by GAM-retained AAV vectors. In summary, this study demonstrates the identification of novel transduction enhancer AAVBlast and the successful development of a gene-activated matrix enabling efficient, localized AAV-mediated gene delivery *in vitro*, providing a promising platform for future GAM applications.

## 1 Introduction

Recombinant Adeno-associated viral (rAAV) vectors are widely recognized as promising tools for efficient delivery of therapeutic transgenes to target cells. Their favorable safety profile—characterized by non-pathogenicity, low immunogenicity, and the ability to mediate long-term transgene expression with only rare integration into host DNA—has established AAV vectors as one of the safest viral gene delivery systems currently available (Moldavskii et al., 2025; Wang et al., 2019; Batty and Lillicrap, 2024). Consequently, AAV vectors have been extensively investigated for the treatment of monogenic diseases, where replacement of a missing or defective gene can restore physiological function. This strategy has led to the successful clinical translation of multiple AAV-based gene therapies, several of which have received FDA approval in recent years (Moldavskii et al., 2025; Zhao et al., 2025; Dunbar et al., 2018). Beyond classical gene replacement approaches, recombinant AAV (rAAV) vectors have gained increasing attention in areas where controlled overexpression of specific genes induces therapeutic effects. In the context of tissue regeneration, rAAV-mediated delivery of growth factors has emerged as a powerful strategy to enhance endogenous repair processes (Cucchiarini et al., 2018). This is particularly relevant for the treatment of large or critical-sized bone defects, which are characterized by impaired bone regeneration and insufficient vascularization (Papachristou et al., 2021; Nauth et al., 2018; Roddy et al., 2018). Numerous studies have demonstrated that local overexpression or sustained delivery of osteogenic and angiogenic factors—such as bone morphogenetic proteins (BMPs) and vascular endothelial growth factor (VEGF) — significantly improves bone regeneration outcomes (Kaspiris et al., 2022; Grosso et al., 2017). This clinical need is underlined by the fact that approximately 10%–15% of the 6.5 million annual bone fractures in the United States result in delayed union or non-union (Peng et al., 2002).

As a result, the application of AAV vectors in regenerative medicine—and particularly in bone regeneration—has gained considerable interest over the past decade. However, despite their advantages, AAV vectors exhibit limitations when administered systemically, including rapid vector clearance and relatively low transduction efficiency at the target site (Moldavskii et al., 2025; Colella et al., 2018). These challenges have prompted the development of alternative delivery strategies aimed at improving local vector retention and enhancing target cell transduction.

One promising approach involves the association of AAV vectors with suitable biomaterials to enable localized and controlled gene transfer. This strategy is particularly attractive in tissue engineering applications, as biomaterials can be modified to actively support tissue regeneration while simultaneously serving as delivery vehicles for gene therapy vectors (Shea et al., 1999; Bonadio et al., 1999). In the field of bone regeneration, a wide range of scaffolding materials has been developed for implantation into bone defects. By integrating AAV vectors into such scaffolds, gene-activated matrices (GAMs) are generated that offer the potential to combine structural support with localized delivery of therapeutic genes, thereby creating a regenerative microenvironment directly at the defect site (D'Mello et al., 2017).

Among biomaterials suitable for bone defect repair, chitosan has gained considerable attention due to its favorable biological and physicochemical properties. Chitosan is a linear polysaccharide derived from chitin, typically obtained from crustaceans or fungi, and is characterized by its biocompatibility, biodegradability, and intrinsic osteoconductive properties (Narayanan et al., 2014; Saravanan et al., 2017). In addition, chitosan-based scaffolds have been shown to support osteoblast adhesion, proliferation, and survival, making them attractive candidates for bone tissue engineering applications (Fakhry et al., 2004). Importantly, incorporation of calcium phosphate ceramics such as β-tricalcium phosphate (β-TCP) into chitosan scaffolds further enhances their suitability for bone regeneration. Composite chitosan/TCP scaffolds exhibit enhanced osteoconductivity, and optimized biodegradation profiles that enable scaffold degradation to occur in parallel with new bone ingrowth, allowing progressive replacement of the scaffold by regenerating bone tissue (Lee et al., 2000; Liu et al., 2013; Azaman et al., 2022). Moreover, the ceramic phase contributes osteoinductive signals and supports osteoblast differentiation and extracellular matrix mineralization, thereby promoting new bone formation (Lee et al., 2000; Yao, 2025). These properties make chitosan/β-TCP composites promising as scaffold materials for gene-activated matrices in bone defect repair.

Beyond the choice of scaffold material and viral vector system, the functionality of viral vectors can be further enhanced by incorporating substances that increase transduction efficiency. In this context, pluronic block copolymers—also known as poloxamers—have emerged as promising modulators of gene transfer. Originally developed to improve drug delivery, early studies already indicated their capacity to enhance viral gene transfer (March et al., 1995; Feldman et al., 1997). Poloxamers are composed of hydrophilic polyethylene oxide (PEO) and hydrophobic polypropylene oxide (PPO) blocks, resulting in amphiphilic molecules whose physicochemical properties can be modified by varying block length and composition. This property confers thermoresponsive behavior, enabling self-assembly into micelles above a critical temperature (Hofig et al., 2012). Initially regarded as passive delivery vehicles, poloxamers are now recognized to actively influence biomaterials and cellular processes. For example, pluronic unimers have been shown to insert into cellular membranes, thereby modulating membrane permeability and the activity of efflux transporters (Kabanov et al., 2002). These membrane-associated effects have been linked to enhanced intracellular uptake of macromolecules, including viral vectors, thereby resulting in increased transduction efficiency (March et al., 1995; Feldman et al., 1997; Hofig et al., 2012). Collectively, these properties render poloxamers attractive candidates for integration into gene-activated matrix (GAM)–based strategies aimed at improving localized viral vector–mediated gene transfer.

Despite extensive research on biomaterials, growth factors, and delivery systems for bone regeneration, gene-activated matrix–based therapies have not yet been successfully translated into clinical practice to replace current gold-standard treatments. Therefore, we investigated a gene-activated matrix to enable efficient and localized AAV-mediated gene delivery for bone regeneration. Importantly, we aimed at developing a workflow that can be easily transferred to other GAM-based strategies. In addition, we identified and characterized the novel poloxamer-based transduction enhancer AAVBlast, that generally improves AAV gene transfer. To our knowledge, this is the first study to investigate a gene-activated matrix based on chitosan/β-TCP scaffolds associated with BMP-2– and VEGF-expressing AAV2 vectors in combination with a poloxamer-based enhancer.

## 2 Materials and methods

If not otherwise stated, chemicals and reagents were obtained from Merck, Darmstadt, Germany.

Synperonic F68 (poloxamer 188) is a commercially available poloxamer (CRODA, Montigny-Le-Bretonneux, France) and was used in this study at a concentration of 20% w/v dissolved in double-distilled water. Poloxamer 111 and 112 are proprietary cationic poloxamers, derived from Synperonic F68 with chemical modifications described in patent application EP3999649A1. Both poloxamers 111 and 112 have been used at a concentration of 20% w/v in double-distilled water.

Mix one and AAVBlast consist of a poloxamer mixture associating either Poloxamer 111 or 112 with a cationic derivative of the commercially available Synperonic F127 (poloxamer 407, CRODA, Montigny-Le-Bretonneux, France), modified according to patent application EP3999649A1. Final concentration of the polymer mixture has been set to 40% w/v in double distilled water, and the ratio between the two components has been carefully adjusted so that the resulting mixture turns into a hydrogel at a temperature close to 37 °C.

All poloxamer solutions have been sterile-filtered with 0.22 µm membranes and tested according to US Pharmacopoeia US Pharmacopoeia (USP) <71> guidelines as well as to be endotoxin‐free (USP <85>) and mycoplasma‐free (USP <63>) (USP (Nonnenmacher and Weber, 2012)).

### 2.1 Cell lines and primary cells

All cells were cultured at 37 °C in a humidified incubator (HERAcell, Thermo Fisher Scientific, Waltham, United States) at 5% CO_2_. HEK293T as well as HeLa cells were cultured in Dulbecco’s modified Eagle medium (DMEM) containing 1% L-alanyl-L-glutamine (Biochrom, Berlin, Germany) and 10% fetal calf serum (FCS; PAN Biotech, Aidenbach, Germany). Saos2 cells were cultured in McCoy’s 5A medium (Gibco/Thermo Fisher Scientific, Waltham, United States) supplemented with 1% L-alanyl-L-glutamine and 15% FCS. HUVEC cells were obtained from PromoCell and cultured in endothelial cell growth medium 2 (PromoCell, Heidelberg, Germany).

Primary human monocyte-derived macrophages were isolated from peripheral blood of two independent healthy donors, according to the protocol approved by the ethics committee at TUM (564/18 S-AS). Whole blood was collected into Falcon tubes, diluted with phosphate-buffered saline (PBS) and layered onto Pancoll human (PAN Biotech) for density gradient centrifugation. Cells were counted and incubated with CD14^+^ magnetic beads (Miltenyi biotec, Bergisch Gladbach, Germany). CD14^+^ cells were isolated using the positive selection program on a Miltenyi autoMACS pro separator (Miltenyi biotec). Purified CD14^+^ monocytes (1.5 × 10^5^ cells) were seeded in Roswell Park Memorial Institute (RPMI; Gibco) medium supplemented with 10% FCS, 1% penicillin–streptomycin, and 50 ng/mL macrophage colony-stimulating factor (M-CSF; Gibco). After 3–4 days, fresh medium was added, and macrophages were used for experiments after 7–9 days of differentiation.

For the collection of bone marrow samples, 3-year-old adult Ripollese sheep (AM Animalia, Spain) were used. All procedures were conducted following Spanish (Real Decreto 53/2013) and European (2010/63/UE) regulations and were approved by the Departament d’Agricultura, Ramaderia, Pesca, Alimentació i Medi Natural of the Catalan Government, under procedure number 11563. Bone marrow was extracted from the sternum of anesthetized animals, previously sedated with 0.5 mg/kg intramuscular midazolam (B. Braun, Melsungen, Germany) and 4 mg/kg intravenous propofol (Baxter, Deerfield, United States). The sternal region was aseptically prepared with alternating scrubs of povidone-iodine (Reig-Jofre, Sant Joan Despi, Spain) and 70% ethanol (Panreac, Castellar del Valles, Spain). The aspiration needle (11-gauge x 11cm; Ranfac, Avon, USA) was inserted perpendicular to the bone until the medullary cavity was accessed. Approximately 8–10 mL of bone marrow was aspirated. Immediately after collection, bone marrow samples were transferred to 75 cm^2^ flasks (Nunc/Thermo Fisher Scientific, Waltham, United States) and DMEM containing 25 mM glucose (Gibco/Thermo Fisher Scientific) supplemented with 10% FCS (Gibco) and antibiotics/antimycotics (PSF, Life Technologies) was added. After 4–5 days in the incubator (Nuaire, Plymouth, United States), supernatant was aspirated and the flasks filled with fresh culture medium before shipment at room temperature in insulated containers.

Alternatively, ovine mesenchymal stromal cells (oMSCs) were isolated from a six-month-old female Merino sheep. Femoral bone was obtained from the Center of Preclinical Research, Technical University of Munich (TUM). Bone marrow was harvested by flushing the femur, and oMSCs were isolated using standard procedures. Following isolation, MSCs were maintained in DMEM (PAN Biotech, Aidenbach, Germany) supplemented with 10% FCS (PAN Biotech), 1% penicillin–streptomycin (Biochrom, Berlin, Germany), 0.1% fungizone (Invitrogen), 1% MEM non-essential amino acids (Invitrogen, Carlsbad, United States), 1% L-alanyl-L-glutamine (Biochrom), 1% sodium pyruvate (Invitrogen), and 2% heat-inactivated sheep serum (Thermo Fisher Scientific). Passaging was with 0.05% Trypsin-EDTA (Invitrogen). oMSC were verified by flow cytometry for absence of CD45 and MHC class II and presence of CD44 using an Attune flow cytometer (Thermo Fisher Scientific). 97% of live cells (7-AAD-negative) (BD Biosciences) were negative for CD45 and of these 100% were positive for CD44 (data not shown).

Tri-lineage differentiation assays (osteogenic, adipogenic, and chondrogenic) were performed using oMSC at passages two to four, according to Heidari et al. (Heidari et al., 2013), with the modifications described below. Osteogenic differentiation was induced by culturing oMSCs for 18 days in DMEM supplemented with 10% FCS, 1% penicillin–streptomycin, 0.1% fungizone, 100 nM dexamethasone, 50 µM ascorbate-2-phosphate, and 10 mM β-glycerophosphate. Medium was changed twice weekly. Mineral deposition was visualized by staining with 2% Alizarin Red S solution. Images were acquired, and bound dye was extracted and quantified as described by Gregory et al. (Gregory et al., 2004).

Adipogenic differentiation was induced for 14 days using DMEM supplemented with 10% FCS, 2% sheep serum, 1% penicillin–streptomycin, 0.1% fungizone, 100 nM dexamethasone, 10 μg/mL insulin, 500 µM 3-isobutyl-1-methylxanthine, and 200 µM indomethacin, with twice-weekly medium changes. Cells were fixed with formalin, washed twice with PBS, and lipid-containing vacuoles were stained with 0.3% Oil Red O solution. After imaging, the dye was extracted using isopropanol and absorbance was measured at 490 nm.

Chondrogenic differentiation was performed using a micro-pellet culture system. Pellets were cultured for 3 weeks in DMEM supplemented with 1% FCS, 1% penicillin–streptomycin, 0.1% fungizone, 100 nM dexamethasone, 50 μg/mL ascorbate-2-phosphate, 40 μg/mL proline, 2 mM sodium pyruvate (Thermo Fisher Scientific), ITS+1 supplement, 10 ng/mL transforming growth factor-β3 (TGF-β3; PeproTech, Rocky Hill, United States), and 200 ng/mL bone morphogenetic protein-2 (BMP-2; PeproTech). Medium was changed three times per week. After differentiation, pellets were washed with PBS, air-dried, and digested overnight at 60 °C in 1 mg/mL papain solution. Sulfated glycosaminoglycan (sGAG) content was quantified using the Blyscan assay (Biocolor, Belfast, Northern Ireland). DNA content was measured using the Quant-iT PicoGreen assay (Invitrogen), and sGAG levels were normalized to DNA content.

### 2.2 Angiogenic tube formation assay

Human umbilical vein endothelial cells (HUVECs) from pooled donors were purchased from PromoCell and maintained in Endothelial Cell Growth Medium 2 (EGM-2; PromoCell) according to the manufacturer’s instructions. For tube formation assays, growth factor–reduced Matrigel (Corning Inc., Corning, United States) was dispensed into 96-well plates (60 µL per well) and allowed to polymerize at 37 °C. Prior to the assay, HUVECs were starved overnight in endothelial cell medium lacking VEGF, basic fibroblast growth factor (bFGF), and insulin-like growth factor 1 (IGF-1). A total of 1,5 × 10^4^ HUVECs in 10 µL were seeded onto each solidified Matrigel bed, followed by the addition of 100 µL conditioned medium. Conditioned medium was generated from HEK293T cells transduced with AAV vectors (MOI 1 × 10^4^ gc/cell) encoding either eGFP or VEGF. Non-transduced HEK293T cells served as negative controls. The medium was replaced with fresh medium after 24 h post-transduction. Supernatants were collected 48 h later, centrifuged and used immediately for tube formation assays. Tube formation was assessed after 3 h of incubation using an inverted microscope (Nikon Eclipse TE2000-S with NIS Elements BR3.1 software). Images were analyzed using ImageJ software (version 1.52a) with the Angiogenesis Analyzer plugin, as previously described (Schneider et al., 2012; Carpentier et al., 2020).

### 2.3 Production and purification of recombinant AAV vectors

cDNA encoding eGFP, human VEGF165 or human BMP-2 was cloned into either a single-stranded AAV transfer vector (pAAV-MCS; Agilent, Santa Clara, United States) or a double-stranded (self-complementary) AAV transfer vector (kindly provided by Prof. Dr. Dirk Grimm, University of Heidelberg). In addition, empty AAV2 vectors were cloned that did not contain any coding sequences. Cloning was performed using standard cloning techniques or sequence- and ligation-independent cloning (SLIC) (Li and Elledge, 2007). In single-stranded vectors, transgene expression was driven by the cytomegalovirus immediate-early (CMVi) promoter, followed by a β-globin intron and the human growth hormone polyadenylation signal (pA). Double-stranded vectors contained the CMVi promoter, followed by a short synthetic intron and the bovine growth hormone pA. All plasmid constructs were verified by sequencing.

Recombinant AAV particles were produced by triple transfection of HEK293T cells with the respective transfer plasmid, the adenoviral helper plasmid pHelper (Agilent), and rep-cap plasmids encoding AAV capsids of serotype 2 (pAAV-RC; Agilent), serotype 6 (pAAV2/6; kindly provided by Prof. Dr. Christian Kuppat, TUM University Hospital), or serotype 8 (pAAV2/8; Penn Vector Core, United States). Viral particles were purified by iodixanol gradient ultracentrifugation (Salk Institute of Biological Sciences protocol). Vector genome titers were determined by quantitative PCR using the AAVpro Titration Kit (qPCR) Ver. 2 (Takara Bio Inc., Kusatsu, Japan) according to the manufacturer’s instructions. Prior to titration, AAV particles were denatured with 2 mM NaOH at 55 °C for 45 min. The reaction was terminated with 2 mM HCl. qPCR was performed using a LightCycler® 480 system (Roche Diagnostics, Basel, Switzerland), with the following cycling conditions: initial denaturation at 95 °C for 2 min, 35 cycles of denaturation at 95 °C for 5 s and annealing/extension at 60 °C for 30 s. A melting curve analysis was included to verify amplification specificity. Purified AAV preparations were aliquoted and stored at −80 °C until use.

### 2.4 AAV transduction and cell lysis

For AAV transduction, the indicated numbers of vector genomes (vg) and the transduction enhancer MG-132 (2 µM) or poloxamers (as indicated, 0.5 µL per well (96-well format) corresponding to a final concentration of 0.4%; developed and provided by OZ Biosciences, Marseille, France) were prepared in 10–20 µL of serum-free medium. Poloxamers were pipetted on ice. Vector and enhancer solutions were combined in equal volumes, resulting in a final transduction volume of 20–40 µL per well. Cells were seeded (1 × 10^4^ cells per well in 96-well plates) for transduction the next day. On the day of transduction, culture medium was removed and transduction mixture added to each well. After 3 h at 37 °C, serum-containing medium was added.

For evaluation of AAV-mediated eGFP expression, cells were washed twice with PBS and lysed with 150 µL lysis buffer (250 mM Tris-HCl, pH 7.8, containing 0.1% Triton X-100). After 20 min at room temperature, lysates were transferred to black, flat-bottom 96-well plates (Corning Costar, Arlington County, United States). eGFP fluorescence was measured using a Tecan Spark 10M multimode plate reader (Tecan, Männedorf, Switzerland) with optimized gain settings, a z-position of 2.8 × 10^4^, and top-reading mode. Autofluorescence from untransduced negative controls was subtracted from AAV-transduced cells for relative fluorescence unit (RFU) correction.

To normalize fluorescence signals, total protein concentration was determined from each lysate using the Bio-Rad protein assay (Bio-Rad Laboratories, Hercules, United States). 10 μL of samples or bovine serum albumin (BSA) standards (1.5 mg/mL serial dilution) were mixed with 150 µL ddH_2_O and 40 µL protein assay dye. Following a 20-min incubation at room temperature, absorbance was measured at 590 nm.

### 2.5 Production of Chitosan/TCP scaffolds

Chitosan/TCP scaffolds were produced as described (Iqbal et al., 2022) except that mushroom derived chitosan was used. In brief, chitosan (Chitolytic, C-M-98-5014,412) was dissolved in 2% acetic acid (v/v) and 0%–30% TCP (Merck, 900205) was added. 100 μL solutions were added to individual wells of 96-well plates. Freeze drying was performed at −80 °C for 24 h, followed by freezing at −100 °C for additional 24 h under vacuum (43 mTorr) resulting in 90% porosity with pore sizes ranging from 20 to 200 µm. Full physicochemical description of the chitosan/β-TCP scaffolds is provided in (Iqbal et al., 2024; Yildizbakan et al., 2024).

### 2.6 Generation and characterization of gene-activated matrices

Gene-activated matrices were generated by direct application of AAV2 vectors onto chitosan/β-tricalcium phosphate (β-TCP) scaffolds. Reporter gene–expressing AAV2 were prepared at the indicated genome copy numbers in 10 µL serum-free DMEM and carefully pipetted onto chitosan/β-TCP scaffolds (1–2 mm thickness), which fully absorbed the applied volume. To assess AAV release kinetics, 100 µL serum-free medium (SFM) was added to each scaffold to ensure complete coverage. Supernatants were collected at the indicated time points and AAV genome copies were quantified by qPCR. At the final time point, a suspension of 5 × 10^4^ oMSCs in 30 µL serum-free advanced DMEM was added to each scaffold.

For growth factor expressing AAV2, oMSC were seeded directly after AAV-loading, except for experiments including AAVBlast. In these experiments, AAV2 vectors were loaded onto chitosan scaffolds and 0.5 or 1 µL AAVBlast was applied subsequently. Prior to oMSC seeding, the poloxamer was allowed to gel for 10 min at 37 °C. After addition of oMSC, scaffolds were incubated for 30 min at 37 °C followed by addition of 100 µL serum-containing advanced DMEM. The final serum concentration was adjusted to 5% FCS for growth factor–expressing AAVs and 10% FCS for reporter gene–expressing AAVs. AAV-mediated transgene expression was assessed either by quantification of eGFP fluorescence using a Tecan Spark 10M multimode plate reader or by using culture supernatants for ELISA analysis.

### 2.7 Growth factor and AAV2 capsid quantification by ELISA

Growth factor concentrations in cell culture supernatants following AAV2 transduction were quantified using Human BMP-2 and Human VEGF DuoSet ELISA kits (R&D Systems, Minneapolis, United States). Wash and dilution buffers, substrate and stop solutions, and white, flat-bottom Costar 96-well plates were obtained from the DuoSet ELISA Ancillary Reagent Kit 2 (R&D Systems). For assessment of AAVBlast-mediated stabilization, AAV2 capsid titers were determined using an AAV2-specific ELISA kit (XpressBio, Frederick, United States), which included pre-coated 96-well plates, antibodies supplied at working concentrations, and wash buffer concentrate. All ELISA assays were performed according to the manufacturers’ instructions. After addition of stop solution, optical density was measured at 450 nm with wavelength correction at 540 nm using a Tecan Spark 10M multimode plate reader. Protein concentrations were calculated by interpolation from standard curves generated using a four-parameter logistic (4 PL) regression model in GraphPad Prism (GraphPad Software, San Diego, United States).

### 2.8 Gene expression analysis

Total RNA was isolated from individual wells using the Single Cell RNA Purification Kit (Norgen Biotek Corp., Thorold, Canada) according to the manufacturer’s instructions for monolayer-cultured cells. For evaluation of intracellular AAV2 DNA levels, DNA was isolated from cells using the DNeasy® Blood & Tissue Kit (QIAGEN, Hilden, Germany) according to the manufacturer’s instructions. RNA as well as DNA concentration and purity were assessed using a NanoDrop spectrophotometer (Thermo Fisher Scientific, Waltham, United States). The isolated DNA was used for quantification of AAV2 genome copies by qPCR, whereas isolated RNA was used for cDNA synthesis and RT-qPCR.

For cDNA synthesis, 50 ng of total RNA per sample was reverse transcribed using the SensiFAST™ cDNA Synthesis Kit (Meridian Bioscience, Cincinnati, United States) following the manufacturer’s protocol. Reactions were assembled in a final volume of 20 µL. Reverse transcription was performed in a thermal block with the following program: 10 min at 25 °C, 15 min at 42 °C, 5 min at 85 °C, and hold at 4 °C. Synthesized cDNA was quantified using a NanoDrop spectrophotometer (Thermo Fisher Scientific) for quality control.

Quantitative real-time PCR was performed using the Takyon™ No Rox SYBR® MasterMix dTTP Blue (Eurogentec, Liège, Belgium) on a Light Cycler 480 (Roche) according to the manufacturer’s instructions. Gene-specific forward and reverse primers (Metabion, Munich, Germany) are listed in Table 1. Thermal cycling conditions were as follows: 50 °C for 2 min, 95 °C for 3 min, 40 cycles at 95 °C for 10 s, 60 °C for 20 s, and 72 °C for 30 s. A melt curve analysis was performed at the end of each run to verify amplification specificity. Gene expression levels were determined relative to the reference gene oGAPDH using the 2^−ΔΔCT^ method.

**TABLE 1:** Primer sequences for gene expression analysis.

Gene	Primer sequence
oGAPDH	Fw: 5′- ATC CTG CCA ACA TCA AGT GG -3′ Rev: 5′- CAG CCT TCT CCA TGG TAG TGA -3′
hBMP-2	Fw: 5′- ATG GAT TCG TGG TGG AAG TGG -3′ Rev: 5′- GTT ACT AGC AAT GGC CTT ATC -3′
hVEGF	Fw: 5′- TGC AGA TTA TGC GGA TCA AAC C -3′ Rev: 5′- TGC ATT CAC ATT TGT TGT GCT GTA G -3′
oRunx2	Fw: 5′- TCG CCT CAC AAA CAA CCA -3′ Rev: 5′- AGG GAC CTG CGG AGA TTA -3′
oOsx	Fw: 5′- CAG CGG CGT GCA GTA AAT -3′ Rev: 5′- CTG GGA ACG AGT GGG AAA A-3′
oOCN	Fw: 5′- GAA GAG ACT CAG GCG CTA CCT -3′ Rev: 5′- CAT CAC AGT CAG GGT TGA GC -3′
oOPN	Fw: 5′- TCC CAC TGA CAT TCC AAC AA-3′ Rev: 5′- CTG TGG CAT CTG GAC TCT CA -3′
eGFP	Fw: 5′- GAC GTA AAC GGC CAC AAG TT -3′ Rev: 5′- GCC GTA GGT CAG GGT GGT -3′

### 2.9 XTT assay

To normalize AAV-mediated growth factor secretion between cells cultured on 3D scaffolds and those growing in monolayer, a XTT assay was performed using the Cell Proliferation Kit II (XTT; Roche, Basel, Switzerland) according to the manufacturer’s instructions. Following incubation for a minimum of 4 h at 37 °C, absorbance was measured at 492 nm with a reference wavelength of 650 nm using a Tecan Spark 10M multimode plate reader (Tecan, Männedorf, Switzerland).

### 2.10 Flow cytometric analysis

AAV2-eGFP-transduced primary monocyte-derived macrophages (MDMs) and Saos-2 cells were transferred to V-bottom 96-well plates using standard procedures. The cells were washed with FACS buffer (1% BSA in PBS) and stained with antibody staining solution containing Live/Dead APC-eFluor™ 780 (1:2000; BD Biosciences, Franklin Lakes, United States), CD14-BV605 (1:200, Biolegend, San Diego, United States) and CD86-Alexa Fluor 700 (1:200; BD Biosciences) diluted in PBS. The cells were fixed with intracellular (IC) fixation buffer (eBioscience/Thermo Fisher Scientific, Waltham; United States). Flow cytometric analysis was performed using an ID7000 flow cytometer (Sony Biotechnology, San Jose, United States) and data were analyzed using FlowJo 10 (FlowJo, LLC, Ashland, United States). eGFP fluorescence was detected in the FITC channel.

### 2.11 Statistical analysis

All data are presented as mean ± standard error of the mean (SEM) and were calculated using descriptive or row statistics as appropriate. Statistical analyses were performed using one-way or two-way analysis of variance (ANOVA). Prior to ANOVA, data were tested for normality and log-normality using the Shapiro–Wilk test. Multiple comparisons were conducted using Dunnett’s *post hoc* test for one-way ANOVA and Sidak’s or Tukey’s multiple comparisons test for two-way ANOVA, as appropriate. Mostly experiments with a minimum of three independent replicates (n ≥ 3) were included in statistical analyses. A p value <0.05 was considered statistically significant, indicated as follows: ns = p ≥ 0.05; * = p < 0.05; ** = p < 0.01; *** = p < 0.001; **** = p < 0.0001. All analyses were conducted using GraphPad Prism 10 (GraphPad Software, San Diego, United States).

## 3 Results

### 3.1 Selection of ssBMP-2 and dsVEGF- expressing AAV2 vectors

In this study, we investigated a gene-activated matrix (GAM) and a novel enhancer of AAV transduction. While the present work was conducted in the context of bone regeneration, the underlying development strategy is broadly applicable to other therapeutic settings in which gene-activated matrices may be employed. The genes of interest for this study were bone morphogenetic protein-2 (BMP-2) and vascular endothelial growth factor (VEGF165), whereas chitosan/tricalcium phosphate scaffolds were selected as the biomaterial for the GAM.

To identify the most efficient AAV serotype for gene delivery in the context of bone regeneration *in vitro*, multiple cell lines were transduced with dsAAV2, dsAAV6, or dsAAV8 vectors encoding eGFP. HeLa and HEK293T cells were used to assess general transduction efficiency, Saos-2 cells served as a context-relevant cell line, and oMSC were used as primary target cells with respect to potential future *in vivo* studies in sheep large bone defect models. Prior to AAV screening, oMSC were evaluated regarding their multilineage differentiation potential by culturing cells in osteogenic, chondrogenic, or adipogenic induction media followed by respective staining. Representative images showed successful osteogenic (Supplementary Figure S1A) and adipogenic differentiation (Supplementary Figure S1B). Quantitative analyses revealed that all oMSC exhibited significantly increased mineralized calcium deposition (osteogenesis), sulfated glycosaminoglycan production (chondrogenesis), and lipid accumulation (adipogenesis), confirming their multipotent capacity (Supplementary Figures S1C–E).

**FIGURE 1:**
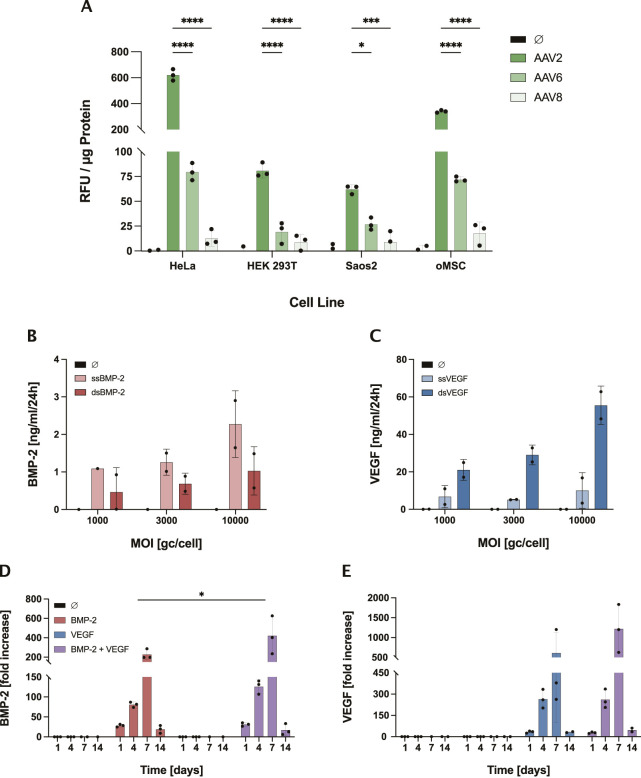
Selection and validation of AAV vectors expressing BMP-2 and VEGF. **(A)** Multiple cell lines were transduced with dsAAV2, dsAAV6, or dsAAV8 vectors encoding eGFP (MOI 1 × 10^4^ gc/cell), and fluorescence of lysed cells was quantified 3 days post-transduction. AAV2 vectors mediated significantly higher levels of reporter gene expression compared to AAV6 and AAV8. **(B,C)** AAV2 vectors encoding BMP-2 or VEGF in single-stranded (ss) or double-stranded (ds) DNA formats were used to transduce primary ovine mesenchymal stromal cells (oMSC) (MOI 1 × 10^4^ gc/cell). MG-132 (2 µM) was used as a transduction enhancer. ELISA analysis 2 days post-transduction revealed the highest secretion levels for ssBMP-2 and dsVEGF. **(D,E)** Selected vector design was evaluated by transduction of oMSC with AAV2 vectors expressing ssBMP-2 or dsVEGF, either individually or in combination, at a MOI of 1,5 × 10^4^ gc/cell/virus. Empty AAV2 vectors were used to add up to a total MOI of 3 × 10^4^ gc/cell in single growth factor virus settings. Growth factor mRNA levels relative to GAPDH were quantified by RT-qPCR over a 2-week period, revealing a peak at day 7. Statistical analyses were performed using n = 3 **(A,D,E)** independent biological replicates.

Three days after AAV transduction of cell lines and primary cells, quantification of eGFP from cell lysates revealed significantly higher expression levels mediated by AAV2 compared with AAV6 and AAV8 across all tested cell types (Figure 1A). Specifically, fluorescence levels with AAV2 were 7.78-fold (HeLa), 4.21-fold (HEK293T), 2.3-fold (Saos-2), and 4.72-fold (oMSC) higher than with AAV6. eGFP expression from AAV6 was 2.15- to 6.23-fold higher than AAV8, which yielded near-background fluorescence. Based on these results, all subsequent experiments were conducted using AAV2 vectors.

Next, the optimal transgene expression cassettes for efficient delivery of human BMP-2 and VEGF were evaluated by comparing single-stranded (ss) and double-stranded (ds) DNA formats. Primary oMSC were transduced with AAV2 at increasing multiplicities of infection (MOIs) in the presence of transduction enhancer MG-132 (Johnson and Samulski, 2009). Quantification of growth factor concentrations in culture supernatants 48 h post-transduction revealed cassette-dependent expression differences. ssBMP-2 consistently yielded higher BMP-2 secretion than dsBMP-2, whereas dsVEGF resulted in markedly higher VEGF levels compared with ssVEGF across all tested MOIs (Figures 1B,C). At an MOI of 10,000, dsBMP-2 produced 1.03 (±0.64) ng/ml/day, compared with 2.27 (±0.89) ng/ml/day for ssBMP-2. In contrast, dsVEGF achieved 55.54 (±10.27) ng/ml/day, whereas ssVEGF reached only 10.05 (±9.54) ng/ml/day. Overall, AAV2-mediated VEGF secretion exceeded BMP-2 production by approximately 10- to 25-fold, depending on vector genome configuration and MOI, consistent with previous reports demonstrating that transgene size and genome structure are critical determinants of AAV-mediated expression efficiency. The larger size of the BMP-2 transgene limits efficient packaging in a double-stranded AAV genome, thereby reducing vector functionality. In contrast, smaller transgenes such as VEGF can be accommodated in a double-stranded configuration, resulting in earlier onset and substantially higher levels of transgene expression (McCarty et al., 2001).

To further characterize these vectors, AAV-mediated gene expression kinetics were assessed in primary oMSC *in vitro*. Cells were transduced with ssBMP-2 and dsVEGF AAV2 vectors either individually or in combination, and relative mRNA expression was quantified over a 14-day period. For both transgenes, expression levels increased gradually between days 1 and 4, peaked at day 7, and declined by day 14 post-transduction, regardless of whether vectors were applied alone or in combination (Figures 1D,E). Notably, co-transduction enhanced growth factor mRNA expression, reaching statistical significance for BMP-2 but not for VEGF. These experiments confirm that ssBMP-2 and dsVEGF expressing AAV2 vectors efficiently deliver bone regeneration-specific growth factor genes to relevant cells *in vitro*.

The biological activity of produced and secreted growth factors was subsequently verified *in vitro*. BMP-2 functionality was assessed using the osteogenic differentiation model cell line ATDC-5, either by direct transduction with AAV2 BMP-2 or by treatment with conditioned supernatant from AAV-transduced oMSC. The produced BMP-2 resulted in enhanced calcium mineralization under induced as well as non-induced conditions (Supplementary Figures S2A,B). In addition, osteogenic gene expression was analyzed in primary oMSC 4 weeks post-transduction. Cells were transduced with each vector individually or in combination. mRNA levels of AAV-mediated BMP-2 and VEGF as well as early osteogenic marker oRunx2 were significantly increased, while the intermediate marker oOsx and late markers oOPN and oOCN displayed increased expression levels, which were not statistically significant (Supplementary Figures S2C–H).

VEGF functionality was verified by tube formation of primary HUVEC cells upon treatment with supernatant from HEK293T cells that were transduced with AAV2 VEGF. Representative images display increased tube formation mediated by AAV2-expressed VEGF (Supplementary Figure S3A). The number of junctions, tubes, and meshes, as well as total tube length was significantly increased by AAV2-mediated VEGF, demonstrating robust pro-angiogenic activity (Supplementary Figure S3B).

### 3.2 Gene-activated matrix: combination of AAV2 vector with chitosan tricalcium phosphate scaffold

**FIGURE 2:**
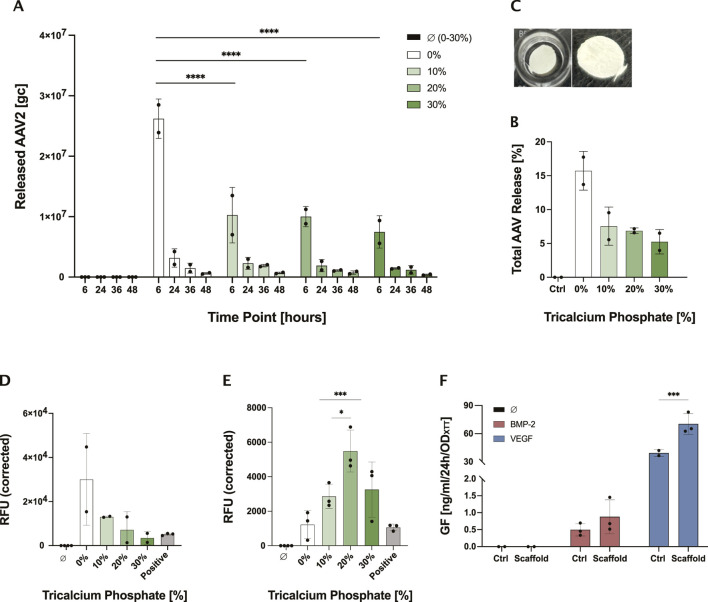
Selection of Chitosan Scaffolds containing 10% Tricalcium Phosphate (TCP). **(A)** Chitosan scaffolds containing 0, 10, 20 or 30% β-TCP were loaded with 2 × 10^8^ genome copies (gc) of eGFP expressing AAV2. Quantification of released AAV genomes by qPCR displayed an initial release that decreased over time. Chitosan/TCP scaffolds that were not loaded with AAV vectors served as negative controls. **(B)** Total release of AAV. Tricalcium phosphate reduced AAV release from approximately15% to ∼5–7%, indicating enhanced AAV binding. **(C)** Representative images of a chitosan/TCP scaffold in 96-well format. **(D)** Infectivity of released AAV2 particles obtained after 6h evaluated by transduction of HEK293T cells. The positive control were cells transduced at a multiplicity of infection (MOI) of 5 × 10^3^ gc/cell. eGFP of cell lysates was quantified after 3 days. **(E)** Retained vectors and *in situ* transduction was assessed by seeding ovine mesenchymal stromal cells (oMSC) onto each scaffold. Highest fluorescence levels 7 days post-seeding were exhibited by scaffolds containing 20% TCP. Ovine MSC directly transduced at an MOI of 1 × 10^4^ gc/cell served as a positive control. **(F)** Chitosan scaffolds containing 10% TCP were loaded with 1 × 10^9^ gc of AAV2 vectors encoding BMP-2 or VEGF and subsequently seeded with oMSC. After 3 weeks, secreted growth factor (GF) levels were quantified by ELISA and normalized to optical density (OD) values from XTT assay. Cells cultured on AAV-loaded scaffolds produced higher growth factor levels compared to cells cultured in AAV-loaded control wells without scaffolds. Statistical analyses were performed using n = 2 **(A,B,D)** or n = 3 **(E,F)** independent biological replicates.

AAV2 vectors were associated with chitosan-based scaffolds to generate gene-activated matrices (GAMs). Chitosan scaffolds containing 0%, 10%, 20%, or 30% tricalcium phosphate (TCP) were evaluated for viral release kinetics (Figure 2A). Each scaffold was loaded with reporter gene–expressing AAV2, and viral release into the supernatant was quantified over 48 h. All scaffold compositions exhibited an initial burst release followed by a gradual decline in AAV release over time. Notably, chitosan scaffolds without TCP released significantly more AAV particles than TCP-containing scaffolds. At 6 h post-loading, chitosan-only scaffolds released 2.62 (±0.33) × 10^7^ genome copies (gc), whereas TCP-containing scaffolds released 0.93 (±0.29) × 10^7^ gc. Following the initial release phase, AAV levels in the supernatant decreased to near-background levels and remained low for at least 1 week for all scaffold compositions (data not shown). Cumulative quantification of AAV revealed that 15.72% (±2.86%) of loaded AAV particles dissociated from pure chitosan scaffolds, but only an average of 6.55% (±1.68%) from TCP-containing scaffolds, indicating increased AAV retention by TCP incorporation (Figure 2B). A representative image of the GAMs is shown in Figure 2C.

Infectivity of released AAV particles collected after 6 h was evaluated by transduction of HEK293T cells. Quantification of eGFP expression 3 days post-transduction corroborated the release data, with the highest fluorescence levels observed for AAV released from chitosan scaffolds lacking TCP (Figure 2D). Increasing tricalcium phosphate proportions led to reduced eGFP fluorescence, indicating lower levels of infectious vector particles in the supernatant. As the majority of AAV particles seemingly remained associated to the scaffolds, primary ovine MSC were seeded directly onto each gene-activated matrix to assess *in situ* transduction and to model cellular infiltration under physiologically relevant conditions. Analysis of eGFP expression 7 days post-seeding confirmed efficient transduction from scaffold-retained vectors. Maximal eGFP levels were detected in oMSC seeded on chitosan scaffolds containing 20% tricalcium phosphate, followed by comparable expression levels for 10% and 30% TCP (Figure 2E). In contrast, chitosan-only scaffolds yielded the lowest eGFP expression. Based on the balance between AAV retention and release, chitosan scaffolds containing 10% TCP were selected for subsequent experiments.

To further validate the gene-activated matrix, primary oMSC were seeded onto chitosan scaffolds containing 10% TCP, loaded with 1 × 10^9^ vector genomes AAV2 encoding BMP-2 or VEGF. Quantification of BMP-2 and VEGF concentrations in cell culture supernatants after 3 weeks confirmed successful *in situ* transduction and growth factor secretion, with 0.89 (±0.50) ng/ml/day/OD_XTT_ BMP-2 and 70.44 (±11.08) ng/ml/day/OD_XTT_ VEGF. Cells seeded on AAV-loaded scaffolds secreted higher levels of growth factor compared with control cells cultured in AAV-loaded wells without scaffolds (Figure 2F), reaching statistical significance for VEGF but not BMP-2.

### 3.3 Identification of the novel poloxamer-based transduction enhancer AAVBlast

**FIGURE 3:**
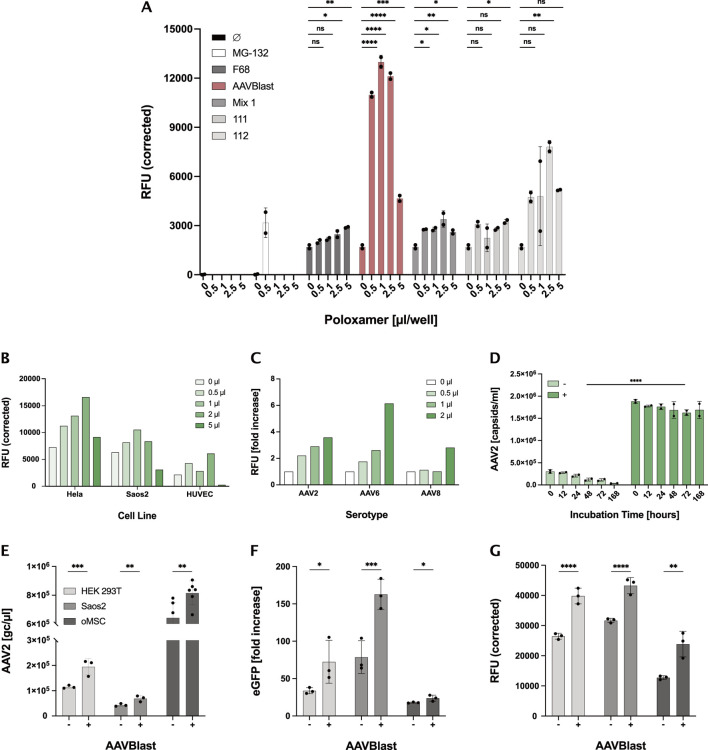
Poloxamer AAVBlast enhances AAV-mediated Gene Transfer. **(A)** Multiple Poloxamers were combined with reporter gene expressing AAV2 vectors and used for transduction of primary ovine mesenchymal stromal cells (oMSC). Proteasome-inhibitor MG-132 (2 µM) served as a positive control. EGFP quantification of cell lysates after 3 days identified AAVBlast as the most potent transduction enhancer providing a significant increase in fluorescence levels. AAVBlast (0.5 µL/well) was tested for transduction enhancement of **(B)** AAV2 in different cell lines (MOI 1 × 10^4^ gc/cell) and of **(C)** several AAV serotypes (MOI 1 × 10^4^ gc/cell) in primary oMSC. **(D)** Stabilization of AAV2 vectors by 0.5 µL AAVBlast per well was evaluated by seeding 2 × 10^9^ gc of AAV into 96-well plate followed by incubation at 37 °C for defined time periods and quantification *via* ELISA. AAVBlast significantly increased levels of detected AAV2. **(E–G)** Cell lines as well as primary cells were transduced with eGFP-expressing AAV2 at an MOI of 1 × 10^4^ gc/cell in presence of 0.5 µL AAVBlast per well. **(E)** Intracellular AAV2 DNA, **(F)** transgene mRNA relative to GAPDH, or **(G)** eGFP-mediated fluorescence was quantified. Poloxamer AAVBlast resulted in a significant increase on all levels of AAV2-mediated transgene expression. Statistical analyses were performed using n = 2 **(A,D)** or n = 3 **(E–G)** or n = 6 (E, oMSC) independent biological replicates.

To optimize AAV-mediated gene transfer, several poloxamer formulations were evaluated for their ability to enhance transduction. Reporter gene–expressing AAV2 vectors were combined with increasing concentrations of proprietary poloxamers and used to transduce primary oMSCs. Quantification of eGFP expression in cell lysates 3 days post-transduction identified Poloxamer AAVBlast as the most effective enhancer, yielding a significant 6.4-fold increase in fluorescence with the lowest AAVBlast concentration used while reaching an optimum at 1 µL (Figure 3A). A similar enhancement could be observed across the cell lines HeLa, HEK293T and HUVEC (Figure 3B) as well as across the AAV serotypes 2, six and 8 (Figure 3C), confirming the broad transduction-enhancing activity of AAVBlast.

To assess a potential vector-stabilizing effect, AAV2 was incubated for varying durations in the presence or absence of the cationic poloxamer at 37 °C. AAVBlast significantly increased detectable AAV capsid levels by 6.2-fold compared with untreated controls (Figure 3D), indicating enhanced vector stability and increased availability of AAV2 particles. Consistent with these findings, AAVBlast treatment resulted in significantly elevated intracellular AAV genome copy numbers, transgene mRNA expression, and AAV-mediated protein levels in HEK293T cells, Saos-2 cells, and primary oMSC (Figures 3E–G).

### 3.4 AAVBlast promotes AAV-mediated osteogenic gene expression

To further validate AAVBlast in a bone regeneration–relevant context, its impact on osteogenic gene expression following AAV2-mediated delivery of BMP-2 and VEGF was examined. Primary ovine MSCs were transduced in the presence or absence of AAVBlast with BMP-2– and VEGF-expressing AAV2 vectors, either individually or in combination. Relative mRNA expression of the transgenes and the osteogenic markers oRunx2, oOsx, oOCN, and oOPN was quantified 4 weeks post-transduction.

**FIGURE 4:**
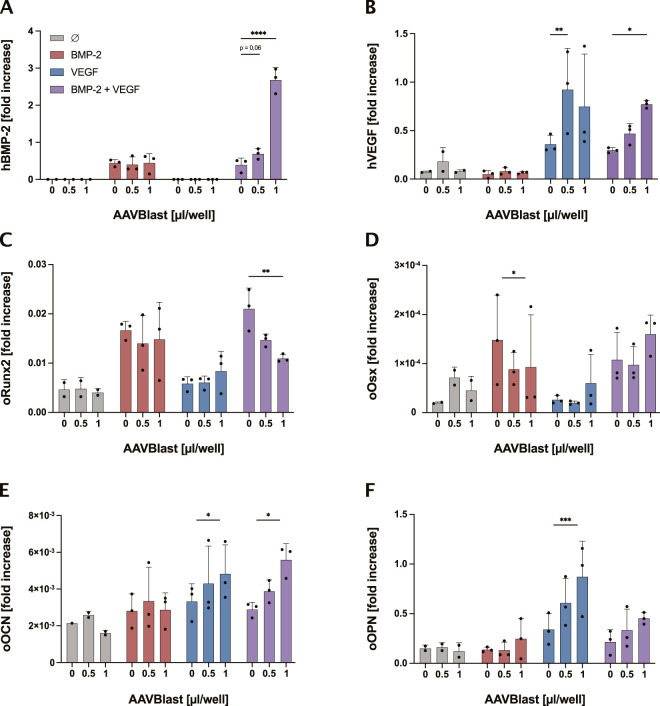
AAVBlast promotes AAV-mediated osteogenic gene expression. Primary ovine mesenchymal stromal cells were transduced with AAV2 vectors expressing BMP-2 and VEGF, separately or in combination, at an MOI of 1 × 10^4^ genome copies per vector/cell in a 96-well plate. Different concentrations of Poloxamer AAVBlast were used for transduction enhancement. Gene expression relative to GAPDH was analyzed 4 weeks post-transduction. The genes investigated were the growth factor genes **(A)** BMP-2 and **(B)** VEGF, the **(C)** early osteogenesis marker Runt-related transcription factor 2 (Runx2), **(D)** intermediate marker Osterix (Osx), and the **(E,F)** late markers Osteocalcin (OCN) and Osteopontin (OPN). The expression of the transgenes and the late osteogenic markers were significantly increased by AAVBlast. The intermediate and early marker genes were decreased by AAVBlast at this time point. Statistical analyses were performed using n = 3 independent biological replicates.

AAVBlast significantly enhanced BMP-2 expression in cells transduced with the combination of AAV2-BMP-2 and AAV2-VEGF vectors (Figure 4A). oMSC transduced with AAV2-VEGF, alone or in combination, displayed ∼2.5-fold increased VEGF mRNA levels in the presence of AAVBlast (Figure 4B). Expression of the early osteogenic marker oRunx2 was increased by AAV2-mediated BMP-2 but was reduced by AAVBlast in cells transduced with the vector mixture (Figure 4C). A similar pattern was observed for the intermediate osteogenic marker oOsx, with elevated expression induced by AAV2-BMP-2 and partial reduction due to AAVBlast treatment (Figure 4D). In contrast, expression of late-stage osteogenic markers was positively affected by AAVBlast. oOCN mRNA levels were elevated following transgene delivery, with a trend toward VEGF-dependent regulation, and were further increased by ∼ 2-fold in the presence of AAVBlast in cells transduced with both vectors (Figure 4E). Similarly, oOPN expression was induced by AAV-mediated VEGF delivery and was further enhanced by AAVBlast treatment (Figure 4F). These results demonstrate that AAVBlast enhances AAV2-mediated delivery of bone regeneration-promoting genes to relevant cells *in vitro*.

### 3.5 Enhancement of gene-activated matrix with poloxamer AAVBlast

**FIGURE 5:**
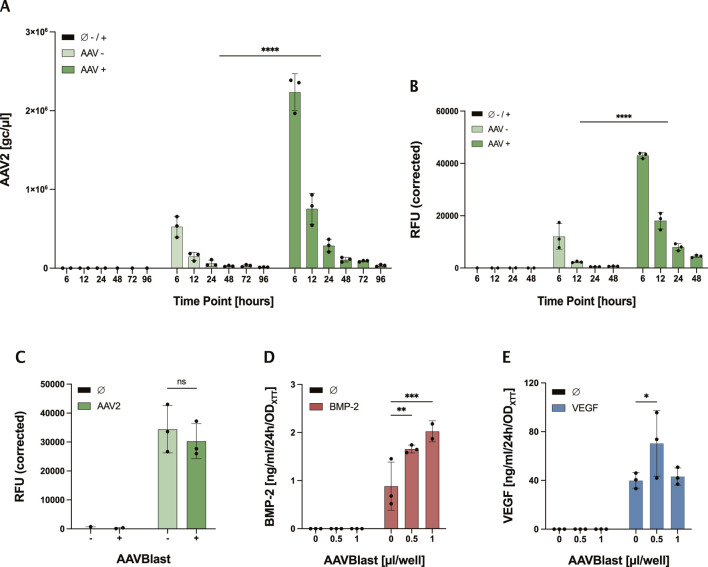
Poloxamer AAVBlast enhances transduction of Gene-Activated Matrix-released AAV2. **(A)** Chitosan/10% tricalcium phosphate (Ch/10% TCP) scaffolds were loaded with 1 × 10^9^ genome copies (gc) of eGFP-expressing AAV2, in presence (+) or absence (−) of 0.5 µL AAVBlast per scaffold. Released AAV particles were quantified at defined time points by qPCR. Significantly higher levels of AAV were detected in the presence of AAVBlast. Scaffolds that were not loaded with AAV vectors served as negative controls. **(B)** The functionality of released AAV2 was assessed by transduction of HEK293T cells, followed by eGFP quantification in cell lysates 3 days post-transduction. AAVBlast significantly increased transgene expression mediated by GAM-released AAV2. **(C)** GAM-retained AAV2 vectors were evaluated by seeding primary ovine mesenchymal stromal cells (oMSCs) onto the scaffolds. eGFP levels in cell lysates 7 days post-seeding were comparable between groups, indicating that AAVBlast primarily stabilizes GAM-released AAV particles while leaving scaffold-retained vectors largely unaffected. **(D,E)** Ch/10% TCP scaffolds were loaded with 1 × 10^9^ gc of growth factor expressing AAV2 in combination with 0, 0.5, or 1 µL Poloxamer AAVBlast, followed by oMSC seeding. Secreted growth factor levels measured by ELISA (normalized to OD from XTT) after 3 weeks were significantly increased in cells transduced in the presence of AAVBlast. Statistical analyses were performed using n = 3 independent biological replicates.

The final step in the development of the gene-activated matrix (GAM) was to evaluate whether AAVBlast enhances transduction mediated by GAM-associated AAV2. eGFP-expressing AAV2 vectors were loaded onto chitosan/10% TCP scaffolds, followed by subsequent application of AAVBlast. Quantification of AAV release over 4 days demonstrated that the poloxamer significantly increased the amount of detectable AAV2 (Figure 5A). The most pronounced effect was observed after 6 h, when the number of detected vector genomes increased 4.2-fold, from 5.3 (±1.3) × 10^5^ to 2.2 (±0.23) × 10^6^ genome copies. Infectivity of scaffold-released AAV2 was evaluated by transduction of HEK293T cells. In line with the release data, cells transduced in the presence of AAVBlast displayed significantly higher eGFP expression compared to released AAV from GAMs lacking AAVBlast (Figure 5B).

In addition, *in situ* transduction by scaffold-retained AAV2 was analyzed by seeding primary oMSC onto the GAMs after completion of the release phase, thereby simulating target cell infiltration under physiologically relevant conditions. Quantification after 1 week revealed a slight, non-significant reduction in eGFP fluorescence mediated by scaffold-retained AAV2 in the presence of AAVBlast (Figure 5C). However, this reduction was less pronounced than would be expected based on the release data alone, suggesting that AAVBlast primarily stabilizes released AAV particles rather than substantially promoting their release from the scaffold.

Finally, the functional performance of the GAM was evaluated using growth factor–expressing AAV2 vectors. Chitosan/10% TCP scaffolds were loaded with AAV2 encoding BMP-2 or VEGF, followed by AAVBlast application and seeding with primary oMSC. Quantification of secreted growth factors confirmed efficient gene delivery by GAM-associated AAV2. Application of 1 µL AAVBlast significantly increased BMP-2 secretion 2.29-fold, to 2.03 (±0.22) ng/mL/24h/OD_XTT_, while 0.5 µL AAVBlast significantly increased VEGF secretion 1.76-fold, to 70.45 (±26.98) ng/mL/24h/OD_XTT_ (Figures 5D,E).

### 3.6 AAVBlast does not enhance transduction of monocyte-derived macrophages with AAV2

It is well established that AAV vectors exhibit limited transduction of immune cells, thereby prolonging the availability of functional AAV particles prior to immune-mediated clearance (Zaiss et al., 2008; Cao et al., 2024; Rossi et al., 2019). In this context, we investigated whether AAVBlast enhances AAV2-mediated transduction of primary monocyte-derived macrophages (MDMs). MDMs were transduced with eGFP-expressing AAV2 vectors at a MOI of 1 × 10^4^ gc/cell in the presence or absence of AAVBlast and analyzed by flow cytometry.

**FIGURE 6:**
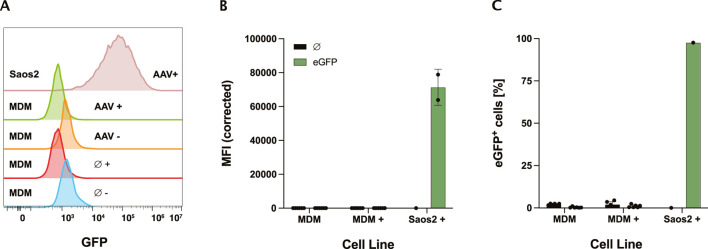
AAVBlast does not enhance Transduction of Monocyte-Derived Macrophages with AAV2. Primary monocyte-derived macrophages (MDMs) were transduced with reporter gene expressing AAV2 at a multiplicity of infection (MOI) of 1 × 10^4^ gc/cell, in the presence (+) or absence (−) of 0.5 µL Poloxamer AAVBlast and analyzed by flow cytometry. **(A)** Representative fluorescence histograms show no difference between non-transduced (∅) and AAV2-treated MDMs. Positive control cell line Saos-2 exhibited robust eGFP expression. **(B)** Quantification of fluorescence intensity and **(C)** percentage of eGFP-positive cells confirm the absence of detectable AAV2-mediated transduction in primary MDMs, irrespective of Poloxamer AAVBlast treatment.

Representative histograms show no detectable difference between AAV2-transduced and non-transduced MDMs, with the exception that AAVBlast alone reduced overall fluorescence intensity (Figure 6A). Quantitative analysis confirmed the absence of detectable AAV-mediated transgene expression in MDMs following AAV2 transduction (Figure 6B). In contrast, Saos2 cells transduced with the same vector dose displayed robust eGFP expression, confirming vector functionality.

Consistent with these findings, the proportion of eGFP-positive MDMs following transduction with AAV2 in the presence of AAVBlast (1.04% ± 0.82%) remained below background levels, as 2.05% (±1.58%) of non-transduced MDMs were gated eGFP-positive (Figure 6C). Together, these results demonstrate that although AAVBlast enhances AAV2 transduction in multiple cell lines and primary target cells, AAV2 remains ineffective at transducing macrophages.

## 4 Discussion

The aim of this study was to develop a gene-activated matrix (GAM) and a novel poloxamer-based enhancer to enable efficient and local AAV gene transfer. While the overall workflow described here is broadly applicable to various GAM-based strategies, the present work focused on the context of bone regeneration, particularly critical-sized bone defects. In an initial screening step, rAAV2 was identified as the most suitable serotype for efficient transduction of relevant target cells *in vitro* (Figure 1A). AAV vectors were selected based on their well-established safety profile, characterized by non-pathogenicity, low immunogenicity, and efficient gene delivery, as well as their ability to mediate long-term transgene expression without integration into the host genome (Moldavskii et al., 2025; Wang et al., 2019).

Evaluation of transgene DNA formats demonstrated that single-stranded BMP-2 and double-stranded VEGF cassettes yielded the most effective transgene expression (Figures 1B,C). These findings are consistent with previous studies showing that double stranded or self-complementary vectors generally mediate a faster onset and higher levels of transgene expression compared with single-stranded AAV vectors by bypassing the rate-limiting step of second-strand synthesis (McCarty et al., 2001). However, the packaging capacity of dsAAV vectors is limited to approximately 2.3 kb, which constrains the efficient encapsidation of larger expression cassettes. The dsBMP-2 sequence used in this study—comprising the inverted terminal repeats (ITRs), CMV immediate-early promoter, synthetic intron, human BMP-2 cDNA, and polyadenylation signal—has a size of ∼2.5 kb. Consequently, the dsBMP-2 genome can most likely not be fully packaged into AAV capsids, leading to reduced vector infectivity, and transgene expression efficiency. In contrast, the smaller VEGF transgene expression cassette is below the size limit of a double-stranded AAV genome, providing a mechanistic explanation for the consistently higher AAV2-mediated VEGF expression observed across all experiments in this study.

Such differences in expression levels may be particularly relevant under *in vivo* conditions, where bone regeneration is a tightly regulated process. Previous work by Peng et al. demonstrated that the ratio of osteogenic and angiogenic signals is critical for achieving synergistic effects during bone healing. In a critical-sized calvarial defect model using lentivirus-transduced muscle-derived stem cells, a fivefold excess of BMP-4 over VEGF resulted in optimal recruitment of mesenchymal stem cells, enhanced cell survival, and increased cartilage formation during early stages of endochondral ossification (Peng et al., 2002). This suggests that the ratio of BMP- and VEGF-mediated signaling critically influences regenerative outcomes. Taken together, these findings indicate that further optimization of the GAM should include evaluation of the ratio between BMP-2- and VEGF-expressing AAV2 vectors. In addition to vector dosing, transcriptional control elements represent an important parameter for fine-tuning transgene expression. In the present study, a cytomegalovirus (CMV) immediate-early promoter was employed to achieve robust growth factor expression. However, for other GAM applications, inducible promoter systems or tissue-specific promoters may provide greater control over transgene expression kinetics, thereby potentially enhancing safety and therapeutic efficacy.

In this study, efficient AAV-mediated transgene expression was confirmed in primary ovine mesenchymal stromal cells (Figures 1D,E), validating the selected vector designs. The biological functionality of rAAV-mediated growth factor expression was demonstrated *in vitro* using established model systems. AAV-mediated BMP-2 expression enhanced calcium mineralization in ATDC-5 cells, while AAV-mediated VEGF expression significantly increased angiogenic tube formation by primary HUVECs, confirming that transgene expression resulted in biologically active proteins that induce relevant downstream effects (Supplementary Figure S2).

The gene-activated matrices (GAMs) evaluated here were generated by loading AAV2 vectors onto chitosan-based scaffolds containing varying proportions of β-tricalcium phosphate (β-TCP). The physicochemical properties of the chitosan/β-TCP scaffolds used in this study have previously been characterized (Iqbal et al., 2024; Yildizbakan et al., 2024). Incorporation of TCP reduced both swelling as well as mass degradation and enhanced cell attachment, particularly in scaffolds containing 10% β-TCP. Functional evidence for successful AAV incorporation was demonstrated by robust transgene expression in cells seeded onto AAV-loaded scaffolds (Figure 2C). In addition, indirect evidence of vector retention was obtained, as only ∼7–16% of the initial vector dose was detected in the supernatant (Figure 2A). Nevertheless, direct characterization of AAV incorporation within the scaffold matrix remains to be addressed in future studies. Although the interaction between AAV vectors and chitosan- or tricalcium phosphate-based scaffolds has been only sparsely investigated, existing reports suggest that chitosan can promote the association of viral vectors for gene delivery (Thomas and Shea, 2013), while calcium phosphate materials have been shown to efficiently bind AAV vectors and enhance AAV-mediated gene transfer *in vivo* (Yang and Chao, 2003). These observations are consistent with our findings and suggest that AAV association with the scaffold is most likely mediated by electrostatic interactions.

Moreover, the stability of AAV vectors within the scaffold represents an important parameter for assessing the suitability of these GAMs for bone regeneration. In this context, robust transgene expression was observed in cells seeded onto the gene-activated matrices up to 1 week after AAV loading and storage at 37 °C (data not shown), suggesting that vector particles remain stable within the scaffold over this period. However, comprehensive evaluation of the stability of scaffold-bound AAV vectors will require further investigation. Taken together, analysis of the time-dependent release profile indicated that the majority of AAV particles remained associated with the chitosan scaffold, with the incorporation of tricalcium phosphate (TCP) further enhancing vector retention (Figure 2A). Based on these findings, chitosan scaffolds containing 10% β-TCP were selected, as they provide a balance between vector retention and release, in combination with the previously reported favorable physicochemical properties and improved cell attachment.

Besides its effects on swelling and scaffold degradation, incorporation of TCP into chitosan scaffolds has been reported to improve osteoconductive properties, thus enabling degradation to occur in parallel with new bone formation, allowing progressive replacement of the scaffold by regenerating bone tissue (Lee et al., 2000; Azaman et al., 2022; Iqbal et al., 2024). Furthermore, chitosan/β-TCP scaffolds have been shown to promote osteoblast proliferation, mineralized matrix deposition, and vascular infiltration both *in vitro* and *in vivo*, indicating that the ceramic phase contributes to enhanced bone regeneration (Azaman et al., 2022; Levengood and Zhang, 2014; Maji et al., 2018). Besides determination of AAV release, infectivity of released as well as scaffold-retained AAV vectors could be verified by successful transduction of HEK293T cells with released vectors and efficient gene transfer to oMSCs seeded onto AAV-loaded scaffolds (Figures 2B,D). These results confirmed the functional integrity of the developed GAM system.

A panel of poloxamers was screened to further enhance AAV mediated gene delivery, leading to the identification of AAVBlast as a potent enhancer of AAV-mediated transduction (Figures 3A–C). Although the precise mechanisms of action of AAVBlast have not yet been elucidated, they likely involve the amphiphilic properties of its poloxamer-based components. The hydrophobic core can induce the insertion of poloxamer unimers into plasma membrane lipid bilayers, thereby decreasing membrane viscosity while increasing lipid exchange (Kabanov et al., 2002). Such membrane remodeling has been shown to promote viral vector transduction. For example, March et al. demonstrated that poloxamer 407 enhances adenoviral gene delivery to vascular smooth muscle cells by maintaining high pericellular vector concentrations (March et al., 1995). Similarly, Höfig et al. reported that the poloxamer Synperonic F108 improves lentiviral transduction in both cell lines and primary cells by acting as a membrane modulator. In that study, increased uptake of propidium iodide as an indicator of enhanced membrane permeability without cytotoxicity implied a reorganization of the membrane microstructure that facilitated viral entry (Hofig et al., 2012). Furthermore, cationic polymers are shown to enhance vector adsorption and transduction *via* charge shielding as they reduce the electrostatic repulsion of negatively charged viral capsids and cell membrane parts that can hinder virus–cell interactions (Davis et al., 2004). Similarly, poloxamer-induced membrane fluidization may increase the accessibility of receptors and reduce steric constraints at the cell surface, thereby further promoting viral adsorption and uptake.

Consistent with these mechanisms, we observed elevated intracellular AAV genome copy numbers, increased transgene mRNA levels, and enhanced protein production upon AAVBlast treatment (Figures 3E–G). Furthermore, the increased stability of AAV particles (Figure 3D) and the higher number of functionally available vectors (Figures 5A,B) by AAVBlast are plausible given the known susceptibility of AAV vectors to aggregation as well as physical or chemical stress (Pathak et al., 2025; Jarand et al., 2024), and the ability of poloxamers to prevent such interactions (Rieser et al., 2022; Strobel et al., 2019; Grossen et al., 2023). These hypotheses are further supported by our observation that co-treatment with the proteasome inhibitor MG-132 synergistically enhanced AAV transduction (data not shown), pointing to complementary effects of AAVBlast on intracellular processing pathways targeted by MG-132. Nevertheless, the relative contributions of increased vector bioavailability, improved AAV stability, and enhanced cellular adsorption and uptake to the overall increase in intracellular AAV DNA remain unclear. Further studies are therefore required to dissect which steps of the transduction pathway are primarily affected by AAVBlast and how these mechanisms collectively contribute to the observed enhancement of AAV-mediated gene delivery.

In addition to stabilizing viral particles, poloxamers have been reported to modulate AAV release and bioavailability when incorporated into biomaterial systems (Madry et al., 2020; Diaz-Rodriguez et al., 2015). Diaz-Rodríguez et al. demonstrated that incorporation of pluronic F-127 into alginate hydrogels enabled efficient encapsulation of rAAV vectors, enhanced rAAV-mediated gene transfer, and allowed for modulation of AAV release depending on hydrogel crosslinking conditions (Diaz-Rodriguez et al., 2015). These findings highlight the capacity of poloxamers to influence release kinetics in matrix-based gene delivery systems. In this study, we evaluated a modular strategy involving the sequential application of AAV and AAVBlast to chitosan/TCP scaffolds. For comparison, a premixed AAV–AAVBlast formulation was also tested but yielded comparable results in terms of AAV release and infectivity (data not shown). The sequential approach was therefore favored due to its simplicity and broader applicability to other GAM-based strategies. As shown in Figure 5A, the addition of AAVBlast increased the number of detected vector particles released from the GAM, which corresponded with enhanced transduction (Figure 5B). However, transgene expression levels in cells transduced by GAM-retained vectors were similar between AAVBlast-treated and untreated conditions (Figure 5C). This suggests that AAVBlast primarily acts on vectors after their release rather than acting on the release itself. Because if the elevated AAV levels in Figure 5A were driven by enhanced vector release, subsequent seeding of cells onto the GAMs would have led to significantly reduced transgene expression levels in AAVBlast-treated GAMs. As this was not observed, we propose that AAVBlast enhances the availability and transduction of GAM-released AAV particles through the mechanisms described above. Nonetheless, the influence of AAVBlast on the binding of AAV to the chitosan/TCP scaffold may still play a role, even though no significant differences in transgene expression were observed. Therefore, further studies are necessary to elucidate the molecular interactions between AAV, chitosan/TCP, and AAVBlast, to distinguish AAVBlast’s enhancing effects from possible influences on vector–scaffold binding.

Moreover, AAVBlast promoted osteogenic gene expression in primary ovine mesenchymal stromal cells (oMSCs) transduced with growth factor–expressing AAV2 vectors. Increased expression was observed for the AAV2-delivered growth factors (Figures 4A,B) as well as for the late osteogenic markers *Ocn* and *Opn* (Figures 4E,F). In contrast, the early osteogenic transcription factors *Runx2* and *Osx* displayed reduced mRNA levels in the presence of AAVBlast (Figures 4C,D). This expression pattern aligns with the relatively late time point of analysis (4 weeks post-transduction), as early osteogenic markers typically peak during early differentiation and decline as osteogenic differentiation progresses, while late markers such as *Ocn* and *Opn* increase at later stages (Lian and Stein, 1992; Stein et al., 2004). Although the gene expression differences observed in this study correlate with AAVBlast treatment, they primarily originate from the downstream-signaling effects of AAV-delivered BMP-2 and VEGF. However, a direct influence of the poloxamer AAVBlast on osteogenic gene regulation cannot be excluded, as pluronics are known to exert biological effects beyond their role as delivery enhancers. As mentioned before, poloxamers exhibit the ability to insert unimers into cellular membranes, thereby altering membrane fluidity, permeability, and downstream signaling pathways (Batrakova and Kabanov, 2008).

Besides, several studies have demonstrated that poloxamer-modified biomaterials can provide a suitable environment for differentiation of mesenchymal stromal cells, thereby promoting *in situ* bone regeneration (Lippens et al., 2010; Diniz et al., 2015; Tateiwa et al., 2021). However, in our study we could not detect differences mediated by AAVBlast regarding osteogenic differentiation of oMSCs (Figure 4 non-transduced), confirming that osteogenic differentiation primarily originates from AAV-delivered osteogenic signals rather than AAVBlast-related effects on mesenchymal stromal cells. Yet, it is important to note that some studies report potentially negative effects of poloxamers on the initial healing steps of bone regeneration as it may push mesenchymal stromal cells towards proliferation instead of differentiation (Park et al., 2021). Our data contradict this observation, which indicates that this effect may be outweighed by the osteogenic induction of the AAV2-delivered growth factors. Nevertheless, this might play a role in a respective *in vivo* setting and thus requires specific attention. As a consequence, investigating the effects of AAVBlast on *in situ* MSC differentiation in the GAM presented in this study requires further investigation.

Finally, we investigated whether AAVBlast alters a key property of AAV vectors that contributes to its favorable safety and immunogenicity profile, namely, their limited transduction efficiency in immune cells (Zaiss et al., 2008; Cao et al., 2024; Rossi et al., 2019; Mingozzi and High, 2013). This aspect is particularly relevant for gene-activated matrix (GAM) applications in bone regeneration, as immune cells—especially monocytes and macrophages—are among the first cell populations to infiltrate a bone defect site (Goodman et al., 2019; Boniakowski et al., 2018). To address this, primary monocyte-derived macrophages were transduced with reporter gene–expressing AAV2 vectors in the presence of AAVBlast. Only background levels of AAV-mediated transgene expression were detected, indicating that AAVBlast does not enhance AAV transduction in these cells. These findings suggest that AAVBlast-mediated transduction enhancement is restricted to cell types that are naturally permissive to AAV infection and does not overcome intrinsic cellular barriers (Nonnenmacher and Weber, 2012), thereby preserving key safety-related properties of AAV vectors. This is particularly relevant for potential *in vivo* applications, where immune cell activation can limit transgene expression and therapeutic efficacy (Rey-Rico et al., 2016; Mingozzi et al., 2007).

Moreover, the restricted infectivity profile of AAV2 vectors may confer an additional level of specificity in the GAM context. Upon implantation into a critical-sized bone defect, a variety of cell types—including immune cells, endothelial cells, and mesenchymal progenitors—infiltrate such a porous scaffold. While immune cells are unlikely to be transduced, resident and infiltrating non-immune cell populations can serve as local producers of therapeutic growth factors such as BMP-2 and VEGF, thereby supporting new bone formation in a spatially confined and physiologically relevant manner.

Over the past decades, extensive efforts have been made towards the development of scaffold-based strategies aimed at restoring bone regeneration in critical-sized bone defects, primarily through the delivery of osteogenic and angiogenic signals. While such approaches have demonstrated considerable promise, accumulating evidence over recent years revealed an additional dimension in bone regeneration that has been overlooked so far: the role of the immune system. In particular, macrophages emerged as key regulators of bone healing, orchestrating the balance between inflammation, tissue regeneration, and remodeling. This led to the concept of osteoimmunomodulation, emphasizing that successful bone regeneration depends not only on osteogenic and angiogenic signaling but also on a finely tuned immune response (Goodman et al., 2019). For example, studies by Xie et al. and Niu et al. demonstrated that macrophage polarization and immune modulation within damaged bone tissue critically determine regenerative outcomes (Xie et al., 2020; Niu et al., 2021).

In the present study, all components of the gene-activated matrix (GAM) were selected, among other properties, based on their favorable safety profiles and low intrinsic immunogenicity, thereby minimizing unintended immune activation. While this strategy supports a permissive regenerative environment, recent evidence suggests that active immunomodulation may further enhance bone repair. Notably, Kim et al. demonstrated that AAV-mediated delivery of immunomodulatory cytokines, such as interleukin-4 (IL-4), can beneficially influence bone regeneration by promoting a pro-regenerative macrophage phenotype (Kim et al., 2025). Conceptually, this approach aligns well with the GAM strategy employed here, as only cells present locally need to be transduced to provide the respective signals. These findings highlight that further optimization of GAM-based approaches should explore the controlled delivery of additional regulatory genes, such as immunomodulatory cytokines like IL-4. Incorporating vectors that express immune-directed genes into the GAM may represent a powerful strategy to further enhance bone regeneration in critical-sized bone defects.

Taken together, the gene-activated matrix (GAM) developed in this study is designed to fulfill multiple functions within a large bone defect. It provides a three-dimensional, porous scaffold that supports cell infiltration and adhesion while serving as a local reservoir for AAV2 vectors encoding BMP-2 and VEGF, thereby enabling localized gene delivery and sustained *in situ* production of osteogenic and angiogenic factors *in vitro*. The modular application of the novel poloxamer-based enhancer AAVBlast further promotes the transduction of GAM-released vectors, improving the gene transfer to surrounding cells and increasing overall production of therapeutic proteins. In addition, prior studies have shown that injectable poloxamer formulations can enhance AAV-mediated gene transfer (Feldman et al., 1997; Madry et al., 2020), and poloxamer-containing matrices have also been investigated in the context of bone regeneration (Diaz-Rodriguez et al., 2015). However, to our knowledge, this is the first study to examine the modular integration of a poloxamer into an AAV-loaded biomaterial scaffold. Our findings demonstrate that this approach has the potential to enhance GAM performance and may represent a broadly applicable strategy. Nevertheless, further work is required to elucidate the molecular interactions between AAVBlast and the individual GAM components, namely, chitosan, β-tricalcium phosphate, and AAV vectors.

Moreover, local retention of AAV vectors within the GAM most likely protects them from rapid degradation and clearance as compared to systemic AAV administration, prolonging their bioavailability and functional activity. The β-tricalcium phosphate (β-TCP) component is also reported to provide osteoconductive properties, potentially supporting directional bone ingrowth and mineralized matrix deposition. However, whether the GAM described here recapitulates these functions under *in vivo* conditions remains to be determined. Future studies will need to address this, including the integration of additional load-bearing structures, as this requirement cannot be met by chitosan–TCP alone. Nevertheless, the combination of these components establishes a pro-regenerative microenvironment that supports angiogenesis and osteogenesis, providing a promising platform for future strategies aimed at enhancing the regeneration of large bone defects.

## Data Availability

The original contributions presented in the study are publicly available. This data can be found here: https://figshare.com/articles/dataset/Gene_expression_data_for_Development_of_a_Gene-Activated_Matrix_for_Enhanced_AAV_Gene_Delivery_i_in_vitro_i_-Musoski_et_al_2026/32536128.
