# Supplementary material for: Development of a gene-activated matrix for enhanced AAV gene delivery in vitro
Source: Front Bioeng Biotechnol. 2026 Jun 10;14:1832901. doi: 10.3389/fbioe.2026.1832901 (PMC13291573; doi:10.3389/fbioe.2026.1832901)
Supplement: Supplementary file 2 [file DataSheet3.pdf]

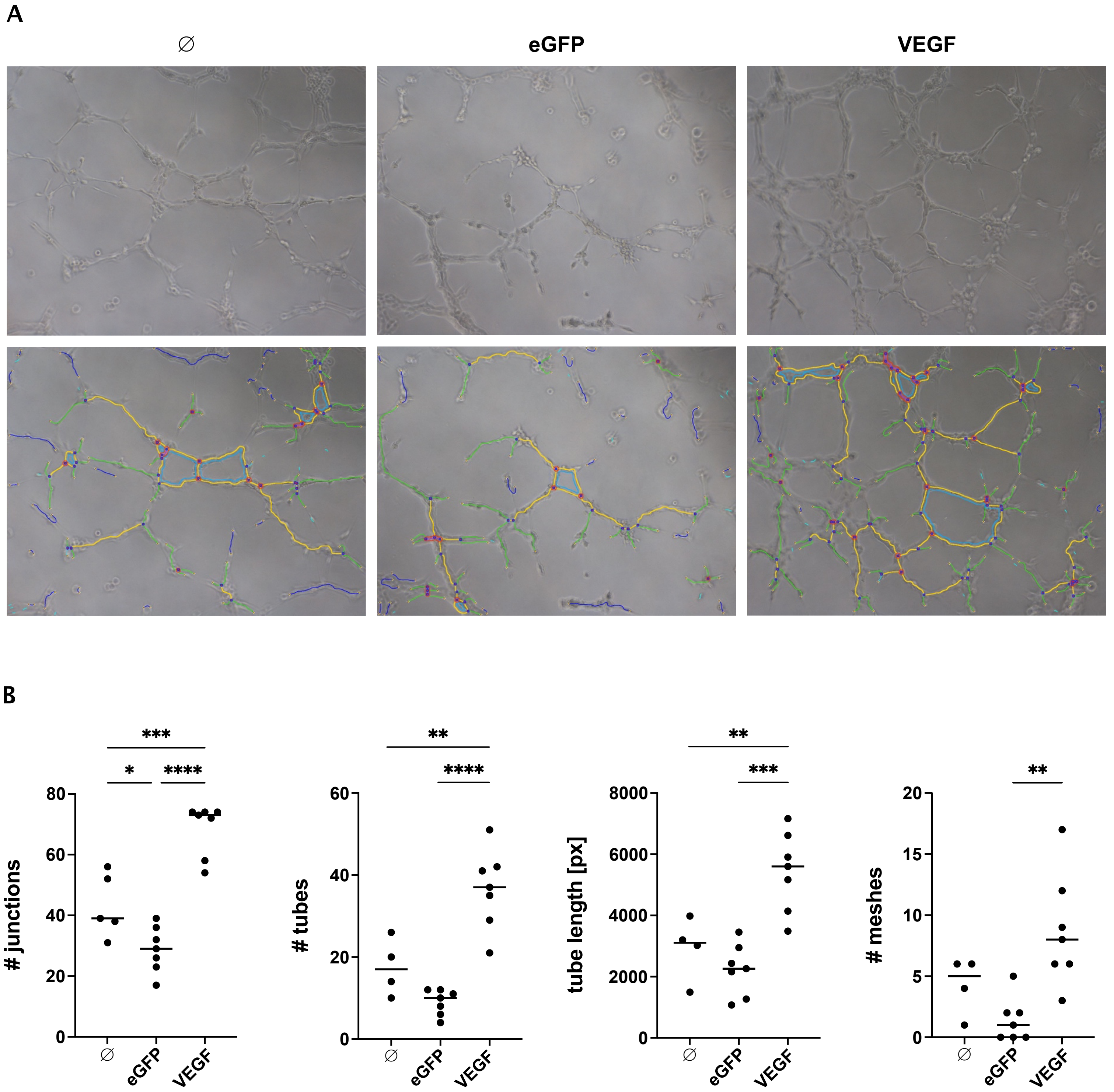

Supplementary Figure S3. AAV2-mediated VEGF enhances tube formation complexity in HUVECs. The biological activity of AAV2-mediated VEGF was assessed using an *in vitro* tube formation assay. HEK293T cells were transduced with VEGF-coding AAV2 vectors (MOI 10000 gc/cell) and conditioned supernatants were applied to Matrigel-embedded human umbilical vein endothelial cells (HUVECs). Tube formation complexity was quantified using the ImageJ software angiogenesis plug-in. HUVECs treated with supernatants from non-transduced or AAV2-eGFP–transduced HEK293T cells served as controls. (**A**) Representative images of HUVEC tube networks with (lower row) and without (upper row) ImageJ-based analysis overlay. (**B**) Quantification of the tube formation parameters number of junctions, number of tubes, total tube length, and number of meshes. AAV2-mediated VEGF expression significantly increased all assessed parameters.
