# Supplementary material for: Development of a gene-activated matrix for enhanced AAV gene delivery in vitro
Source: Front Bioeng Biotechnol. 2026 Jun 10;14:1832901. doi: 10.3389/fbioe.2026.1832901 (PMC13291573; doi:10.3389/fbioe.2026.1832901)
Supplement: Supplementary file 3 [file DataSheet1.pdf]

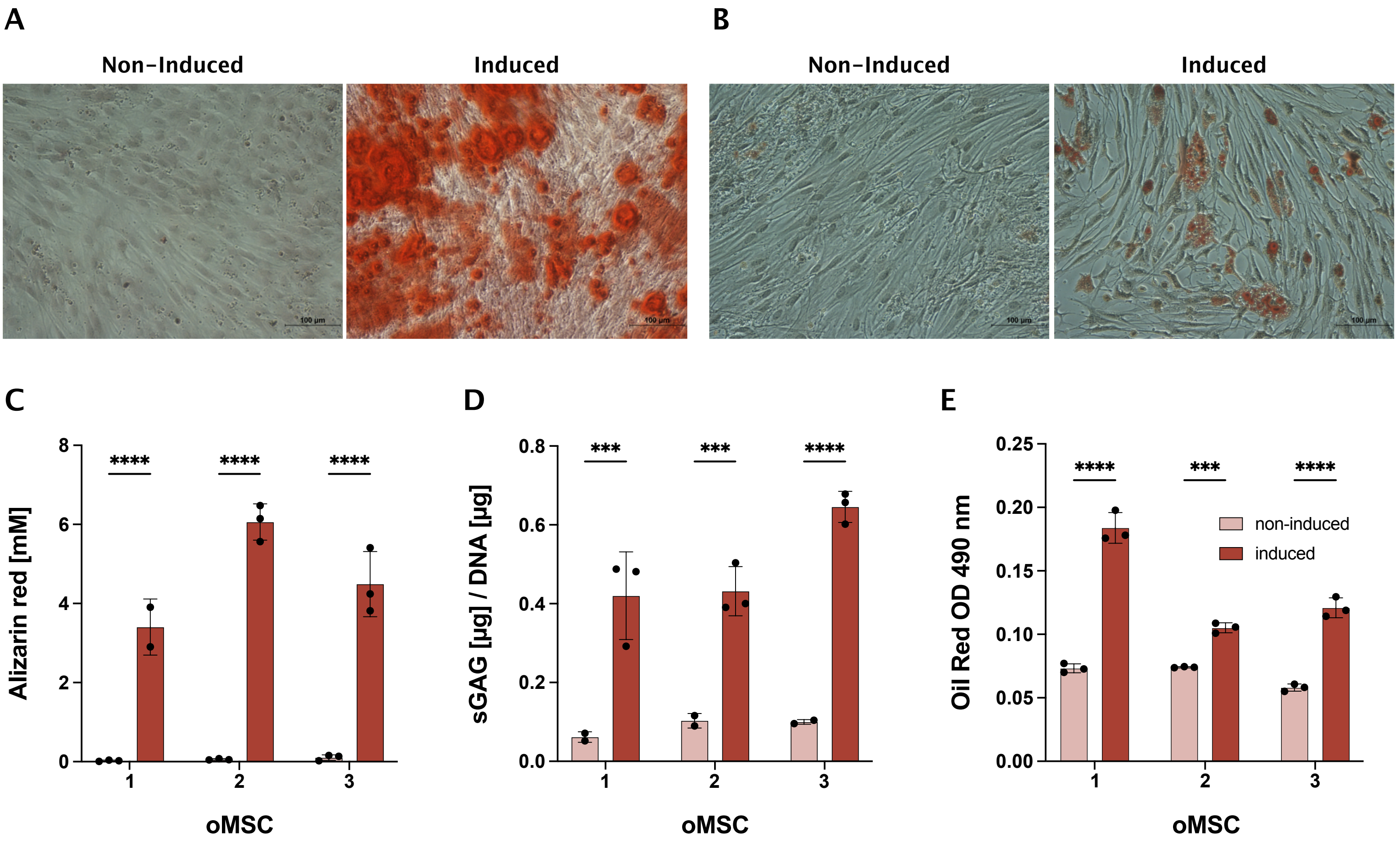

Supplementary Figure S1. Osteogenic, chondrogenic and adipogenic differentiation potential of primary ovine mesenchymal stromal cells (oMSC)

Primary MSC were isolated from sheep (animals 1-3) and tested for their potential to undergo osteogenic, chondrogenic and adipogenic differentiation. Representative images of (**A**) osteogenic and (**B**) adipogenic differentiation. Quantification of osteogenic (**C**), chondrogenic (**D**) and adipogenic (**E**) differentiation of oMSC. All parameters were significantly increased when cultured in the respective differentiation medium, demonstrating the multipotent differentiation potential of the primary oMSC used in this study.
