# Supplementary material for: Development of a gene-activated matrix for enhanced AAV gene delivery in vitro
Source: Front Bioeng Biotechnol. 2026 Jun 10;14:1832901. doi: 10.3389/fbioe.2026.1832901 (PMC13291573; doi:10.3389/fbioe.2026.1832901)
Supplement: Supplementary file 1 [file DataSheet2.pdf]

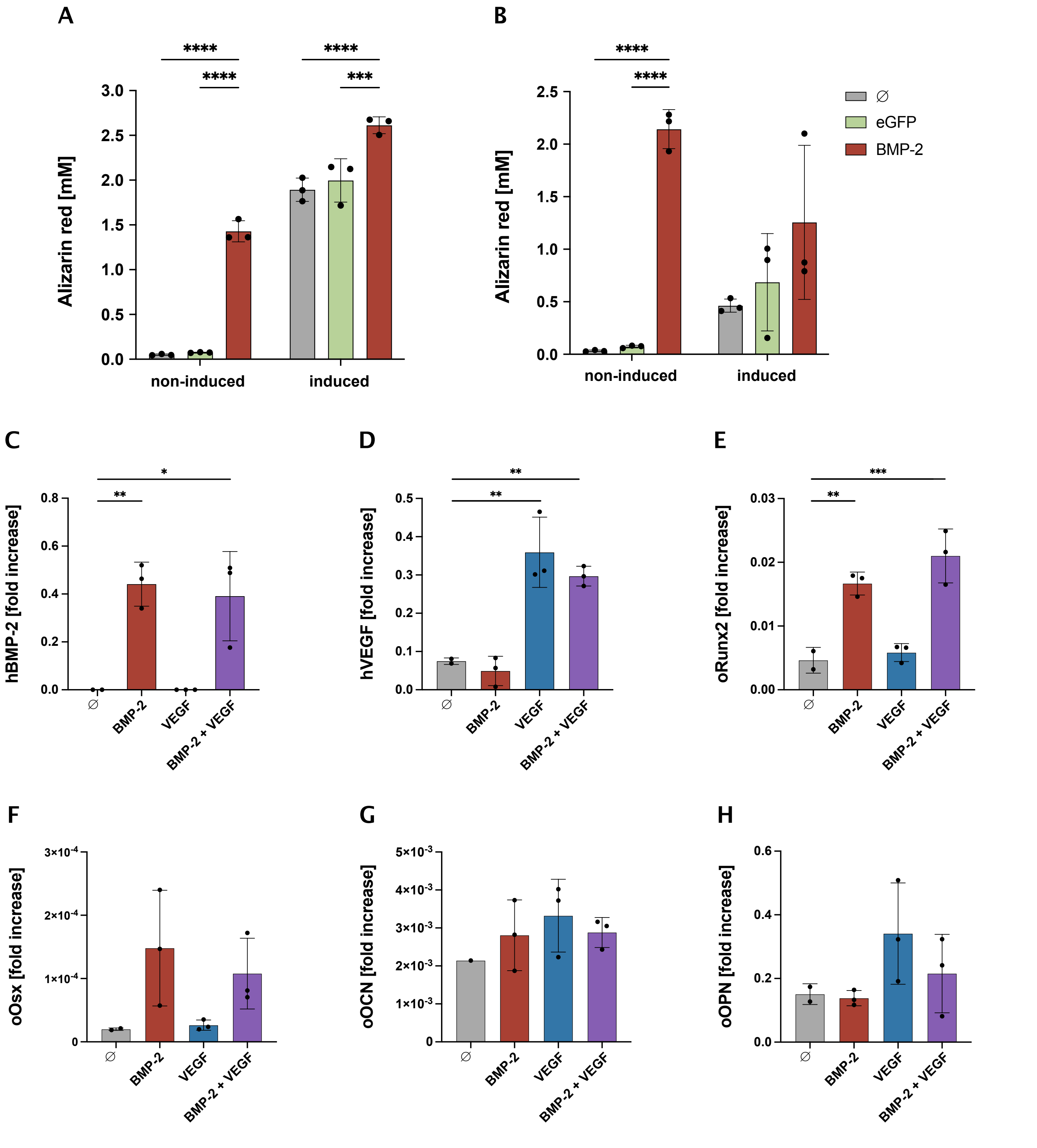

Supplementary Figure S2. AAV-mediated BMP-2 enhances calcium mineralization in ATDC-5 cells and induces osteogenic gene expression in oMSCs.
(**A, B**) Calcium mineralization of murine chondrogenic ATDC-5 cells was evaluated by Alizarin Red staining after 14 days. Cells were cultured in inducing or non-inducing osteogenic differentiation medium. Controls were non-transduced and AAV2-eGFP transduced cells. (**A**) ATDC-5 cells were infected with AAV2-BMP-2 at a multiplicity of infection (MOI) of 20,000 gc/cell. (**B**) Primary ovine mesenchymal stromal cells (oMSCs) were transduced with AAV2 vectors encoding BMP-2 (MOI 10,000 gc/cell). oMSC supernatants were added to ATDC-5 cells cultured with inducing or non-inducing medium. AAV-mediated BMP-2 expression significantly increased Alizarin Red staining intensity. (**C–H**) Primary oMSCs were infected with AAV2-BMP-2 and AAV2-VEGF, individually or in combination, at an MOI of 15,000 genome copies per vector/cell. mRNA expression levels relative to GAPDH were quantified by RT-qPCR after 4 weeks. Expression of the transgenes (**C**) BMP-2 and (**D**) VEGF, as well as (**E**) the early osteogenic marker Runx2, (**F**) the intermediate marker Osx, and (**G, H**) the late osteogenic markers OCN and OPN was analyzed.
